# Prevalence of Exclusive Breastfeeding Practices and associated factors among mothers in Bahir Dar city, Northwest Ethiopia: a community based cross-sectional study

**DOI:** 10.1186/1746-4358-8-14

**Published:** 2013-10-23

**Authors:** Abdulbasit Musa Seid, Melkie Edris Yesuf, Digsu Negese Koye

**Affiliations:** 1 Department of Midwifery, College of Health and Medical Sciences, Haramaya University, Harar, Ethiopia; 2 Department of Human Nutrition, Institute of Public Health, College of Medicine and Health Sciences, University of Gondar, Gondar, Ethiopia; 3 Department of Epidemiology and Biostatistics, Institute of Public Health, College of Medicine and Health Sciences, University of Gondar, Gondar, Ethiopia

**Keywords:** Prevalence, Exclusive breastfeeding, Associated factors, Northwest Ethiopia

## Abstract

**Background:**

Breastfeeding is an unequalled way of providing ideal food for the healthy growth and development of infants. World Health Organization (WHO) recommend exclusive breastfeeding (EBF) for six months which has a great contribution in reducing under five mortality, which otherwise leads to death of 88/1000 live birth yearly in Ethiopia. Hence, this study aimed to assess prevalence of EBF and associated factors in mothers in the city of Bahir Dar, Northwest Ethiopia.

**Methods:**

A community-based cross-sectional study was conducted from 10 to 25 June 2012 among mothers who delivered 12 months earlier in Bahir Dar city, Northwest Ethiopia. A cluster sampling technique was used to select a sample of 819 participants. Data were collected using a structured and pre-tested questionnaire by face-to-face interview technique. Bivariate and multivariate analyses were performed to check associations and control confounding.

**Results:**

Of 819 mother-infant pairs sampled, the overall age appropriate rate of EBF practice was found to be 50.3%. Having a young infant aged 0-1 month (AOR = 3.77, 95% CI = 1.54, 9.24) and 2-3 months (AOR = 2.80, 95% CI = 1.71, 4.58), being a housewife (AOR = 2.16, 95% CI = 1.48, 3.16), having prenatal EBF plan (AOR = 3.75, 95% CI = 2.21, 6.37), delivering at a health facility (AOR = 3.02, 95% CI = 1.55, 5.89), giving birth vaginally (AOR = 2.33, 95% CI = 1.40, 3.87) and receiving infant feeding counseling/advice (AOR = 5.20, 95% CI = 2.13, 12.68) were found to be significantly associated with EBF practice.

**Conclusion:**

Prevalence of exclusive breastfeeding was low in Bahir Dar. Strengthening infant feeding advice/counseling both at the community and institutional levels, promoting institutional delivery, providing adequate pain relief and early assistance for mothers who gave birth by caesarean section, and enabling every mother a prenatal EBF plan during antenatal care were recommended in order to increase the proportion of women practicing EBF.

## Background

Breastfeeding is an unequalled way of providing ideal food for the healthy growth and development of infants [1]. As per the WHO recommendation, infants should receive exclusive breastfeeding (EBF) for the first six months of life to achieve optimal growth, development and health [2-4]. Infants exclusively breastfed for 6 months, presented with fewer infectious episodes such as acute respiratory infection, acute otitis media, and gastroenteritis than their partially breastfed or non breastfed peers [5].

Malnutrition has been responsible, directly or indirectly, for 60% of the 10.9 million deaths annually among children under five worldwide. Over two-thirds of these deaths, which are often associated with inappropriate feeding practices, occur during the first year of life. Globally, no more than 35% of infants are exclusively breastfed during the first four months of life [1]. Global risk assessment of suboptimal breastfeeding indicates that 96% of all infant deaths in developing countries are attributable to inappropriate feeding occurring during the first six months of life [6].

In low income countries like Ethiopia, it has been estimated that practising EBF can reduce under five mortality by 13% [7]. To strengthen the effort in reducing child mortality, the Ethiopian Ministry of Health had targeted an increase in the proportion of exclusively breastfed infants under age 6 months to 70 percent by 2015 as one strategy to improve child health [8]. The 2011 Ethiopian Demographic and Health Survey (EDHS) showed the proportion of infants under six months who received EBF as 52% [9] which improved slightly (only 3%) compared to 2005 EDHS [10]. Therefore, assessing factors associated with exclusive breastfeeding is crucial to implement interventions that speed up the government efforts and decrease the rates and burden of infant morbidity and mortality.

## Methods

A community based cross-sectional study was carried out from 10 to 25 June 2012 among mothers who delivered 12 months before the study began in the city of Bahir Dar, Northwest Ethiopia. Bahir Dar, the capital of Amhara Regional State, is located 565 km Northwest of Addis Ababa, the capital of Ethiopia. In Bahir Dar, there are 9 kebeles – the smallest administrative unit. Based on the 2007 National population census, the city has a total of 180,094 (87,089 males and 93,005 females) population. Out of these, 39,800 were females in the reproductive age groups (15-49 years). In the city, the expected number of pregnancies in a year is 6,843 with the expected live births of 6,483. Number of households in the city are 37,519 [11].

To select study participants, a cluster sampling technique was used. Four kebeles were selected from 9 kebeles in the city using a simple random sampling technique. The sample size was determined by using a single population proportion formula considering the following assumptions: Proportion of EBF 49% taken from 2005 EDHS [10]; 95% level of confidence (Z = 1.96); 5% marginal error. The final sample size was adjusted using the design effect of 2% and 5% non-response rate. Thus, the sample size required was 809 participants, but with all participants in each cluster included, a final sample size of 819 was achieved.

Data on socio-demographic information, obstetric factors like parity, child and breastfeeding practice factors, maternal knowledge about breastfeeding were collected using a pre-tested and structured questionnaire. Data were collected through face-to-face interviews after training both data collectors and supervisors. Data were entered into Epi Info version 3.5.1 and exported to SPSS version 20.0 software package for analysis. The results were presented in the form of tables, figures and text using frequencies and summary statistics such as mean, standard deviation, and percentage to describe the study population in relation to relevant variables. The data were analyzed using logistic regression to determine the effect of various factors on the outcome variable and to control confounding. Most of the variables were fitted to the bivariate logistic regression. Then all variables having a p value ≤ 0.2 in the bivariate analysis were further entered into multivariate logistic regression model. In the multivariate analysis, standard enter techniques were fitted. Variables having p value ≤ 0.05 in the multivariate analysis were taken as significant predictors. Crude and adjusted odds ratios with their 95% confidence intervals were calculated. The Hosmer and Lemeshow goodness-of-fit test was used to assess whether the necessary assumptions for the application of multiple logistic regression were fulfilled and p value > 0.05 was considered a good fit.

Exclusive breastfeeding was defined as giving the infant no other food or drink – not even water – except breast milk, but allowing the infant to receive oral rehydration salt, drops and syrups (vitamins, minerals and medicines). Early initiation of breastfeeding was also defined as the proportion of children who were put to the breast within one hour of birth [1]. Since we included infants less than 12 months, the prevalence of exclusive breastfeeding was determined by using since birth dietary recall method. In this method, the participants were asked if any liquid or food item had been given to the infant, and if so, when that was done for the first time. This also allows to avoid overestimation in determining prevalence. The prevalence is based on women who had babies in each age group at the time of interview. The prevalence of EBF was calculated based on the respective age of the infants. But for infants of 6 months and above, for example, if the infant age was 7 months and the women practised EBF only for the first three months, we did not consider it as the infant received EBF. To say that the infant received EBF, he/she must have received EBF for the first six months for those aged six months or more.

Ethical clearance was obtained from institutional review board of College of Medicine and Health Sciences, University of Gondar (Ref. No: MIDW/964/06/04). A formal letter of cooperation was written to Bahir Dar city health office and the respective kebeles. Voluntary verbal consent was obtained from each study participant.

## Results

### Socio-demographic characteristics of the participants

All 819 mothers in the selected clusters were included in the analysis giving a response rate of 100%. The mean age of the mothers was 26.1 years (SD ± 4.5 years) whereas the median age of the infants was 7 months (IQR = 5 months). A majority, 748 (91.3%) of the mothers were married, 628 (76.7%) were Orthodox-Christian by religion, 801 (97.8%) were Amhara by ethnicity and 493 (60.2%) were house wife by occupation. Two hundred sixty one (31.9%) and 177 (21.6%) of the mothers and fathers did not attend formal education respectively. Regarding average monthly income of the participants, 214 (26.1%) of the households have a monthly income of less than 700 Ethiopian Birr. Seven hundred forty nine (91.5%) of the participants have radio or television in their house. Almost all, 809 (98.8%) of the participants did not smoke cigarettes.

Regarding infants, 445 (54.3%) were female, 360 (44%) were first born and 536 (65.4%) were six months of age or more (Table 1).

**Table 1:** Socio-demographic characteristics of the mothers with their infants (n=819) among mother who gave birth in the last 12 months in Bahir Dar city, Northwest Ethiopia, June 2012

**Variables**	**Frequency (%)**
**Maternal age (in year)**	
17-19	19 (2.3)
20-34	751 (91.7)
35+	49 (6.0)
**Infants age (in months)**	
0-1	31 (3.8)
2-3	113 (13.8)
4-5	139 (17.0)
6 and above	536 (65.4)
**Sex of the infants**	
Male	445 (54.3)
Female	374 (45.7)
**Birth order of the infants**	
First born	360 (44.0)
2^nd^ to 4^th^	438 (53.5)
5^th^ and above	21 (2.6)
**Marital status**	
Married	748 (91.3)
Divorced	34 (4.2)
Widowed/single/separated	37 (4.5)
**Religion**	
Orthodox	628 (76.7)
Muslim	178 (21.7)
Protestant/Adventist	13 (1.6)
**Ethnicity**	
Amhara	801 (97.8)
Others (Agaw, Tigrea, Oromo)	18 (2.2)
**Occupation of the mothers**	
Housewife	493 (60.2)
Government employer	150 (18.3)
Private work	62 (7.6)
Daily laborer	101 (12.3)
Others (farmer/students)	13 (1.6)
**Maternal education**	
No formal education	261 (31.9)
Primary education	161 (19.7)
Secondary education	198 (24.2)
Tertiary education	199 (24.3)
**Husband’s education**	
No formal education	177 (21.6)
Primary education	149 (18.2)
Secondary education	204 (24.9)
Tertiary education	289 (35.3)
**Average monthly income (in Birr)**	
100-700	214 (26.1)
701-1200	207 (25.3)
1201-2000	200 (24.4)
2000-20,000	198 (24.2)
**Access to media (radio or TV)**	
Yes	749 (91.5)
No	70 (8.5)
**Maternal smoking**	
Yes	10 (1.2)
No	809 (98.8)

### Obstetric characteristics of the participants

Of all participants, more than half (55.9%) were multiparous. Most of the respondents, (94.9%) had ANC follow up with a mean number of ANC visits of 3.7 (SD ± 0.7) but 184 (23.6%) were not counseled on breastfeeding during their ANC follow up. Seven hundred and three (85.8%) mothers reported that they had planned during their last pregnancy to provide exclusive breastfeeding. With regards to the place of delivery, 742 (90.6%) delivered their youngest child at health institutions. Seven hundred and twenty eight (88.9%) of the mothers delivered vaginally, and 611 (82.3%) of the mother who delivered at health institutions were counseled on breastfeeding (Table 2).

**Table 2:** Obstetric characteristics of participants (n=819) in Bahir Dar city, Northwest Ethiopia, June 2011

**Variable**	**Frequency (%)**
**Parity**	
Primiparous	361 (44.1)
Multiparous	458 (55.9)
**Presence of ANC**	
Yes	777 (94.9)
No	42 (5.1)
**Frequency of ANC (N=777)**	
1-3 visits	240 (30.9)
4 visits and above	537 (69.1)
**Counseling on BF at ANC (N=777)**	
Yes	593 (76.4)
No	184 (23.6)
**EBF plan during pregnancy**	
Yes	703 (85.8)
No	116 (14.2)
**Delivery place**	
Health facility	742 (90.6)
Home	77 (9.4)
**BF counseling at health facility during delivery (N = 742)**	
Yes	611 (82.3)
No	131 (17.7)
**Mode of delivery**	
Vaginal delivery	728 (88.9)
Caesarean section delivery	91 (11.1)

### Breastfeeding practices

Almost all 815 (99.5%) children had ever breastfed at some point in the past. Of those who had ever breastfed, 709 (87.0%) of the mothers initiated breastfeeding within one hour of birth, 679 (83.3%) had fed colostrum and 220 (27.0%) of mothers gave one or more prelacteal feed. Ninety seven (11.9%) of the participants reported having breast related problems that created difficulty in feeding their infants. Seven hundred and sixty (93.5%) of the participants received infant feeding counseling/advice from different sources such as health professionals other than health extension workers 704 (91.1%), media 321 (41.91%), health extension workers 24 (3.13%), and family members 28 (3.59%). The prevalence of EBF computed using since birth dietary recall method, showed 412 (50.3%) of the participants practised EBF appropriate to their age. The mean duration of EBF was 3.0 months (SD±2.4) (Table 3).

**Table 3:** Breastfeeding practices and knowledge of the respondents (n = 819) in Bahir Dar city, Northwest Ethiopia, June 2012

**Variables**	**Frequency (%)**
**Ever breastfeeding**	
Yes	815 (99.5)
No	4 (0.5)
**Initiation time (n=815)**	
Within one hour of birth	709 (87.0)
After one of birth	106 (13.0)
**Colostrum discarded (n = 815)**	
Yes	136 (16.7)
No	679 (83.3)
**Prelacteal feeding (n = 815)**	
Yes	220 (27.0)
No	595 (73.0)
**Exclusive breastfeeding practice**	
Yes	412 (50.3)
No	407 (49.7)
**Breastfeeding difficulty**	
Yes	97 (11.9)
No	718 (89.1)
**Breast milk adequacy information (n = 815)**	
Yes	473 (58.04)
No	342 (41.96)
**Received infant feeding advice/counseling**	
Yes	766 (93.5)
No	53 (6.5)

**Figure 1:**
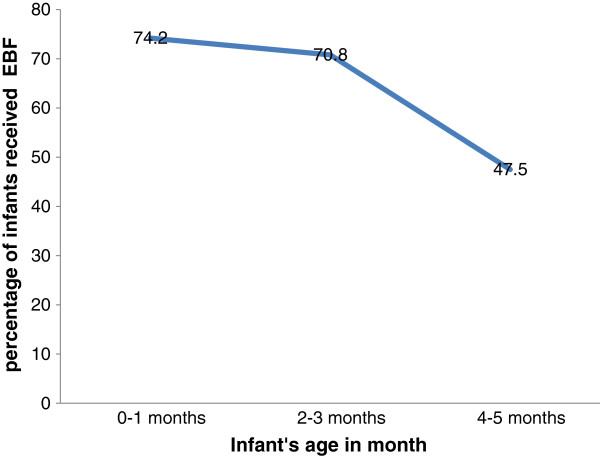
Trends of exclusive breastfeeding practice in infants less than six months of age using since birth dietary recall method among mothers who gave birth within the last six months in Bahir Dar city, Northwest Ethiopia.

Of 283 mothers with infants aged less than 6 months during the study, 169 (59.7%) were exclusively breastfeeding their infants, but only 243 (45.3%) of infants six months and above received EBF when asked retrospectively. In the first month of life, 74.2% of infants received EBF which declined to 70.8% in 2-3 months and 47.5% by the age of 4-5 months (Figure 1).

### Factors associated with exclusive breastfeeding practice

In bivariate analysis the factors found to be significantly associated with EBF were: infant age, occupation, and educational status of the mother, monthly income, husband's educational status, ANC visit during last pregnancy, prenatal EBF plan, place of delivery, mode of delivery, colostrum feeding, and receiving counseling/advice on infant feeding. From these variables, child age, maternal occupation, prenatal EBF plan, place of delivery, mode of delivery and receiving counseling/advice on infant feeding were significantly and independently associated with the EBF practice in multiple logistic regression analysis.

Children with an age interval of 0-1 month and 2-3 months had an adjusted odds ratio of about 3.8 (AOR = 3.77, 95% CI = 1.54, 9.24) and 2.8 respectively (AOR = 2.80, 95% CI = 1.71, 4.58) of receiving EBF compared to children aged 6 months and above. Regarding maternal occupation, housewives had an adjusted odds of 2.2 (AOR = 2.16, 95% CI = 1.48, 3.16) of practising EBF compared to other occupations. Mothers who planned to provide EBF during their last pregnancy had an adjusted odds of 3.8 (AOR = 3.75, 95% CI = 2.21, 6.37) of practising EBF compared to those who did not plan EBF. Regarding the place of delivery, mothers who delivered their last child at a health facility had an adjusted odds 3 to practise EBF compared to those who delivered at home (AOR = 3.02, 95% CI = 1.55, 5.89); whereas those mothers who gave birth vaginally had an adjusted odds of 2.3 (AOR = 2.33, 95% CI = 1.40, 3.87) of practising EBF. Mothers who received counseling/advice on infant feeding had an adjusted odds of 5.2 (AOR = 5.20, 95% CI = 2.13, 12.68) of practising EBF than those who had not (Table 4).

**Table 4:** Bivariate and multivariate analysis of factors associated with exclusive breastfeeding practices among mothers who gave birth in the last 12 months in Bahir Dar city, June 2012

**Variable**	** EBF practice**		**Crude odds ratio (95% CI)**	**Adjusted odds ratio (95% CI)**
	**Yes n (%)**	**No n (%)**		
**Child age**				
0-1 months	23 (74.2)	8 (25.8)	3.47 (1.52, 7.89)	3.77 (1.54, 9.24)*
2-3 months	80 (70.8)	33 (29.2)	2.92 (1.88, 4.54)	2.80 (1.71, 4.58)*
4-5 months	66 (47.5)	73 (52.5)	1.09 (0.75, 1.58)	1.03 (0.68, 1.55)
6 months and above	243 (45.3)	293 (54.7)	1	1
**Maternal occupation**				
Housewife	292 (59.2)	201 (40.8)	2.49 (1.87, 3.33)	2.16 (1.48, 3.16)*
Other occupations	120 (36.8)	206 (63.2)	1	1
**Maternal education**				
No formal education	120 (46.0)	141 (54.0)	1	
Primary education	97 (60.2)	64 (39.8)	1.78 (1.12, 2.65)	
Secondary education	105 (53.0)	93 (47.0)	1.33 (0.92, 1.92)	
Tertiary education	90 (45.2)	109 (54.8)	0.97 (0.67, .41)	
**Husband’s education**				
No formal education	99 (55.9)	78 (44.1)	1	
Primary education	76 (51.0)	73 (49.0)	0.84 (0.58, 1.22)	
Secondary education	118 (57.8)	86 (42.2)	1.11 (0.75, 1.65)	
Tertiary education	140 (48.4)	149 (51.6)	1.46 (1.02, 2.10)	
**Average family income (in Birr)**				
100-700	91 (42.5)	123 (57.5)	1	
701-1200	119 (57.5)	88 (42.5)	1.83 (1.24, 2.69)	
1201-2000	109 (54.5)	91 (45.5)	1.62 (1.20, 2.39)	
2000-20,000	93 (47.0)	105 (53)	1.20 (0.81, 1.77)	
**Smoking cigarettes**				
Yes	3 (30.0)	7 (70.0)	1	
No	409 (50.6)	400 (49.4)	2.39 (0.61, 9.29)	
**ANC visit**				
Yes	405 (52.1)	372 (47.9)	5.44 (2.39, 12.40)	
No	7 (16.7)	35 (83.3)	1	
**Prenatal EBF plan**				
Yes	390 (55.5)	313 (44.5)	5.32 (3.27, 8.67)	3.75 (2.21, 6.37)*
No	22 (19.0)	94 (81.0)	1	1
**Place of delivery**				
Health facility	395 (53.2)	347 (46.8)	4.02 (2.30, 7.02)	3.02 (1.55, 5.89)*
Home	17 (22.1)	60 (77.9)	1	1
**Mode of delivery**				
Vaginal	382 (52.5)	346 (47.5)	2.25 (1.42, 3.56)	2.33 (1.40,3.87)*
Caesarean section	30 (33.0)	61 (67.0)	1	
**Colostrum discarded**				
Yes	56 (41.2)	80 (58.8)	1	
No	356 (52.4)	323 (47.6)	1.58 (1.08, 2.29)	
**Advised/counseled on infant feeding**				
Yes	405 (53.1)	357 (46.9)	7.46 (3.32, 16.72)	5.20 (2.13, 12.68)*
No	7 (13.2)	46 (86.8)	1	

*Significant at p-value of ≤ 0.05.

## Discussion

Exclusive breastfeeding for the first six months is identified as one of interventions to reduce infant morbidity and mortality. Exclusively breastfed children have a much lower risk of infectious diseases than infants who receive other foods [5].

In this study, the rate of exclusive breastfeeding appropriate to the infant’s age was found to be 50.3%. This finding was similar to the 2011 EDHS report (52%) [9], and a study conducted in Ghana (51.6%) [12], but less than the study done in the community assessment finding by Essential Service for Health in Ethiopia (ESHE) in Amhara (87%), Oromiya (79%) and Southern Nation, Nationalities and Peoples Region (66%) [13]. This might be due to the fact that the city of Bahir Dar is not an area of focus for ESHE -the nongovernmental organization that works with local health offices on EBF employed as one strategy to reduce child mortality. However, the findings of this study illustrated higher rates than that of a study conducted in five East and South East Asian Countries (pooled EBF proportion of 35.8%), Malaysia (43.1%) and Nigeria (16.4%) [14-16]. Cross-cultural differences in breastfeeding practices may be part of the explanation.

When asked retrospectively, women with infants aged six months or more reported that only 45.3% had received EBF for six months. This finding was lower than studies done in Cambodia (51.3%), India (61.5%), Iran (56.4%), and Tanzania (58%) [17-20] but higher than that of Canada (13.8%), Thailand (11%), Saudi Arabia (12.2%), Egypt (9.7%) and Kenya (2%) [21-25]. Again cross–cultural differences in breastfeeding practices are likely. The other possible explanation for the variation in EBF practice found in different studies may be the different methods used for computing EBF. In this study we have used since birth dietary recall method which is not standard methods of computing EBF. Many studies such as Ghanaian study [12] showed a significant difference in determining EBF by 24-hour recall methods (a method of computing EBF by asking the respondents to recall what was offered to infants within the last 24 hours preceding the interview) and since birth dietary recall methods (70.2% versus 51.6%).

In this study child age, maternal occupation, prenatal EBF plan, place of delivery, mode of delivery and receiving counseling/advice on infant feeding were significantly and independently associated with the EBF practice in multiple logistic regression analysis.

This study revealed that child age was significantly associated with EBF practice. Infants in age group of 0-1 month and 2-3 months had an odds ratio of 3.8 and 2.8 respectively to receive EBF than whose ages were six months and above. This finding was consistent with the analysis of demographic health survey of Nigeria and Ethiopia [16,26]. The possible explanations for this might be due to the fact that during this period there is a traditional postpartum rest that restricts women from working outside their home, which facilitates and creates favorable conditions for breastfeeding. Another explanation may be due to the short maternity leave in Ethiopia for government employers. There might be also short birth intervals that force the mother to discontinue EBF early.

In this study, housewives were more likely to practice EBF than any other occupations. This finding was consistent with the study done in Saudi Arabia [23], and similar to Canadian [21] and Malaysian [15] studies which showed a positive association between non-working mothers and EBF practice. This might be due to the fact that housewife mothers get to stay longer with their newborn so they may also breastfeed their newborn.

Prenatal EBF plan was also found to be one of the predictors of EBF practice. Mothers who had planned to provide EBF for their last child during pregnancy had an odds ratio of 3.8 to practise EBF compared to those who had not. This finding was in agreement with a study from Cambodia [17]. This might be attributable to planning, increased preparedness, and commitment to achieve EBF.

Place of delivery was one of the predictors of EBF practice. Mothers who delivered their last child at health facility were more likely to practise EBF compared to those who delivered at home. This result was consistent with other studies from Ghana, India, and Tanzania [12,18,20]. This might be due to the postpartum breastfeeding counseling and support provided at the health facility as part of discharge practices. In addition, mothers who gave birth vaginally were more likely to practise EBF than those who gave birth by caesarean section. This finding was consistent with a study from Canada [21].This might be due to the fact that caesarean section related pain and discomfort may prevent mothers from practising EBF.

Receiving infant feeding counseling/advice was also associated with EBF practice. Those mothers who received EBF counseling/advice were more likely to practise EBF than those who did not. Similar results were found by the study conducted in India [18]. This suggests that counseling/advice is effective in improving maternal knowledge and facilitates breastfeeding.

### Limitations

Since we have used the dietary recall since birth method, we might have introduced recall bias, as the mothers might not have recalled accurately when they introduced a food item.

This may under- or over-estimate the true prevalence of EBF. It is also difficult to establish a temporal relationship as the study design was cross-sectional. Despite these limitations, the findings from this study will contribute to the understanding of the factors associated with the EBF practice in the study area.

## Conclusions

Prevalence of exclusive breastfeeding was low in the city of Bahir Dar. Being a housewife, a young infant age, having a prenatal EBF plan, delivering at a health institution, delivering vaginally and receiving counseling/advice on infant feeding were significantly associated with EBF practice. Strengthening infant feeding advice/counseling both at the community and institutional levels, promoting institutional delivery, providing adequate pain relief and early assistance for mothers who gave birth by caesarean section, and enabling every mother to have a prenatal EBF plan during antenatal care were recommended in order to increase the proportion of women practicing EBF.

## Abbreviations

EBF: Exclusive breastfeeding; EDHS: Ethiopian Demographic and Health Survey; ESHE: Essential Service for Health in Ethiopia; WHO: World Health Organization.

## Competing interests

The authors declare that they have no competing interests.

## Authors’ contributions

AM designed the study, performed the statistical analysis and drafted the manuscript. ME and DN participated in the study design, implementation of the study, and contributed to the draft manuscript. All authors contributed to the data analysis, read and approved the final manuscript.
